# Role of frustrations in cell reprogramming

**DOI:** 10.1093/pnasnexus/pgaf303

**Published:** 2025-09-22

**Authors:** Yuxiang Yao, Jieying Zhu, Wenfei Li, Duanqing Pei

**Affiliations:** Laboratory of Cell Fate Control, School of Life Sciences, Westlake University, Hangzhou 310030, China; Westlake Laboratory of Life Sciences and Biomedicine, Hangzhou 310024, China; CAS Key Laboratory of Regenerative Biology, Center for Cell Lineage and Development, Guangzhou Institutes of Biomedicine and Health, Chinese Academy of Sciences, Guangzhou 510530, China; Department of Physics, National Laboratory of Solid State Microstructure, Nanjing University, Nanjing 210093, China; Laboratory of Cell Fate Control, School of Life Sciences, Westlake University, Hangzhou 310030, China; Westlake Laboratory of Life Sciences and Biomedicine, Hangzhou 310024, China

**Keywords:** cell reprogramming, frustration, genetic networks, cell fate

## Abstract

The cell fate transition is a fundamental characteristic of living organisms. By introducing external perturbations, it is possible to artificially intervene in cell fate and trigger cell reprogramming. Revealing the general principle underlying the induced phenotypic reshaping of cell populations remains a central focus in the field of cell biology. In this study, we investigate the energetic and dynamic features of induced cell phenotypic transition from differentiated somatic state to pluripotent state by constructing a Boolean genetic network model. The simulation and experimental results highlight the critical role of genetic frustration in initiating cell fate transitions, although the two ending phenotypic states are typically featured by minimal frustration. In addition, the altered gene expression profiles exhibit a scale-free distribution, suggesting that there exist a small number of critical genes responsible for the cell fate transition. This study provides important insights into the dynamic principles governing effective cell reprogramming caused by artificial or exogenous interventions.

Significance Statement Successful cell reprogramming depends on appropriately controlled culturing and induction conditions. A deep understanding of the biophysical principles underlying cell reprogramming is curial for effectively guiding these processes. We demonstrate that increasing local frustration within the gene regulatory network by inducing factors is critical for reprogramming cell fates, although stable cell states themselves often exhibit minimal frustration. The genetic alterations driven by such genetic frustration display scale-free patterns, suggesting that only a small number of critical genes are responsible for cell fate transitions. Our work establishes genetic frustration as a pivotal driver of reprogramming, revealing a fundamental mechanism for cell fate control and a strategic principle for designing more efficient induction protocols.

## Introduction

Cell fate transitions occur frequently in organisms, driven by programmed regulation of gene expression at multiple levels (1). Unveiling the general principles of cell fate transitions is among the key aspects for understanding the nature of life. Typical examples of cell fate transitions include embryonic development and somatic cell reprogramming. Unlike embryonic development, which typically follows well-established roadmaps, somatic cell reprogramming is often subject to extrinsic interference (2). By simply introducing a few transcription factors (TF) (3–12) under appropriate conditions (13–18), it is possible to convert the cell state of mouse embryonic fibroblasts (MEFs) into that of induced pluripotent stem cells (iPSCs). Breaking existing genetic patterns of cells and rebuilding targeted ones are pivotal steps for the cell reprogramming.

The canonical Waddington epigenetic landscape paradigm elucidates that genetic circuits, external conditions, and noises collectively determine the valleys and ridges on the genetic topographic maps, which represent cell states and barriers of fate transitions, respectively (19–22). Current theoretical models of cell reprogramming involving iPSCs can reasonably describe transition trajectories, predict candidate factors, and even propose controlling strategies (23–27). These theoretical works provide valuable regulatory guidelines for the inducing processes. However, many key aspects on the induced phenotypic reshaping of cell populations is still not fully understood. For instance, why do specific factors contribute to reprogramming rather than others? Is there any common feature in the genetic regulatory network for the productive cell reprogramming events?

In a previous work by Font-Clos et al. (28), a Boolean network model based on experimental data was constructed and utilized to explore the cell state transition between epithelial and mesenchymal states. They elucidated high cell phenotypic plasticity with significant population of metastable hybrid cell states along the phenotypic landscape. In a recent work, Tripathi et al. (29) demonstrated that the gene regulation network determining cell fates are minimally frustrated in the steady cell state, which is the key feature distinguishing biological regulatory networks from random networks. More recently, Wang et al. (30) revealed a concerted mechanism of cell phenotypic transitions by analyzing single-cell RNA sequencing (scRNA-seq) data. Inspired by these works, we further explore the interaction and dynamic features essential for successful cell fate transitions by investigating somatic cell reprogramming processes using a Boolean modeling framework of gene regulation.

Our numerical results and sequencing data reveal that cells leverage local frustration in their genetic network to facilitate productive fate transitions under external interference, despite the global gene regulation network being minimally frustrated. External interference specifically introduces local frustration to the gene nodes involving activation/inactivation transitions, which triggers the re-establishment of cellular order and ultimately alters cell fates. This local frustration not only decreases the barrier height along a pseudo-energy landscape during the fate transition but also shifts the location of the barrier (31). There exists small number of critical genes which are responsible for the cell fate transitions. Our results shed important insights into the general principle of induced cell fate transitions.

## Dynamic framework of cell reprogramming

Experiments reveal that genetic changes during reprogramming typically exhibit a binary-opposite pattern (3–5). Boolean networks (BNs), which inherently have binary features, can be well served as the dynamic model framework to describe the cell reprogramming. BNs have been utilized in the analysis of biosystems for a long time (32–34). In this model, Boolean values 1 and 0 represent the active and silent states of genes, respectively. Consequently, a binary vector ($\vec{x}\in \{0,1{\}}^{N}$) characterizes the state of a genetic network comprising *N* components. BNs abstract regulatory relations as Boolean functions and network topological structures, effectively catching the essential features of the systems but avoiding parametric explosion. The dynamic rules of system can be described as (35),


(1) $$x_{i}(t+1)=\left\{ \begin{array}{cc} 1, & \Sigma_{j}A_{ij}x_{j}>0\\ x_{i}(t), & \Sigma_{j}A_{ij}x_{j}=0\\ 0, & \Sigma_{j}A_{ij}x_{j}<0. \end{array}\right.$$

**Fig. 1.**
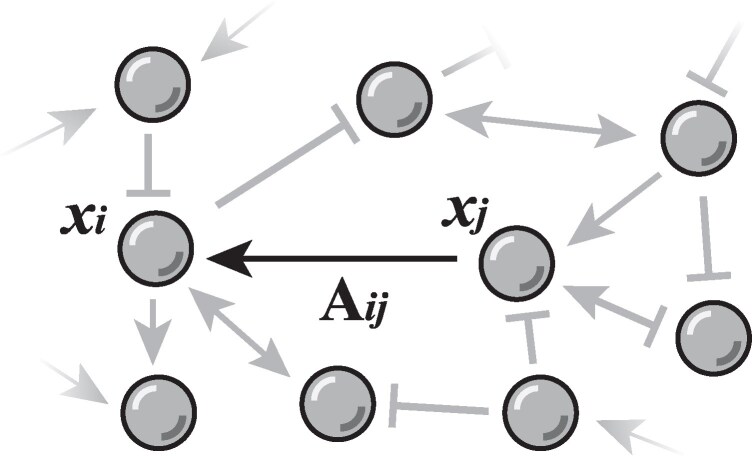
Schematic diagram of the gene regulation network. $A_{ij}$ denotes the regulatory relationship from $x_{j}$ to $x_{i}$. Arrows and T-shaped arrows represent activation and inhibitory regulation, respectively.

Where $A_{ij}=\pm 1$ represents the activation or inhibition operation from gene *j* to gene *i*. The summation runs over all the other genes with a direct regulatory relation with the gene *i* (Fig. 1).

The system evolution employs an asynchronous rule, wherein only one component is randomly selected to update its state at each time step. $\vec{x}(t+1)=\vec{x}(t)$ indicates that the system has reached a steady state. The matrix $\left\{A_{ij}\right\}$, which describes the regulatory relation of different genes, was extracted from experimental data on somatic cell reprogramming reported in the literature. The gene regulatory network contains 88 genetic components and 387 regulatory edges. More detailed settings and the network structure can be found in the Supplementary material.

## Results

### Genetic pseudo-energy landscape along 2D essential space

To elucidate the cellular properties, we employed the two types of cell phenotypes involved in reprogramming. Induced cells gradually lose their differentiated characteristics and re-establish pluripotent potential, corresponding to a transition from somatic state (S) to pluripotent state (P). Moreover, the cell morphology often undergoes a transition from a mesenchymal-like (M) state to an epithelial-like (E) state as featured by weaker migration capacity. Thus, one cell’s phenotypic feature can be quantified by two phenotypic scores $\Phi_{PS}$ and $\Phi_{EM}$, respectively. The phenotypic scores are defined by


(2) $$\Phi_{i}=\sum_{\;j\in \{\phi_{i}^{+}\}}x_{j}-\sum_{k\in \{\phi_{i}^{-}\}}x_{k},$$

where $\left\{\phi_{i}^{+}\right\}$ and $\left\{\phi_{i}^{-}\right\}$ respectively denote positive and negative marker genes of the phenotype *i* (i=PS or EM) (see Methods).

**Fig. 2.**
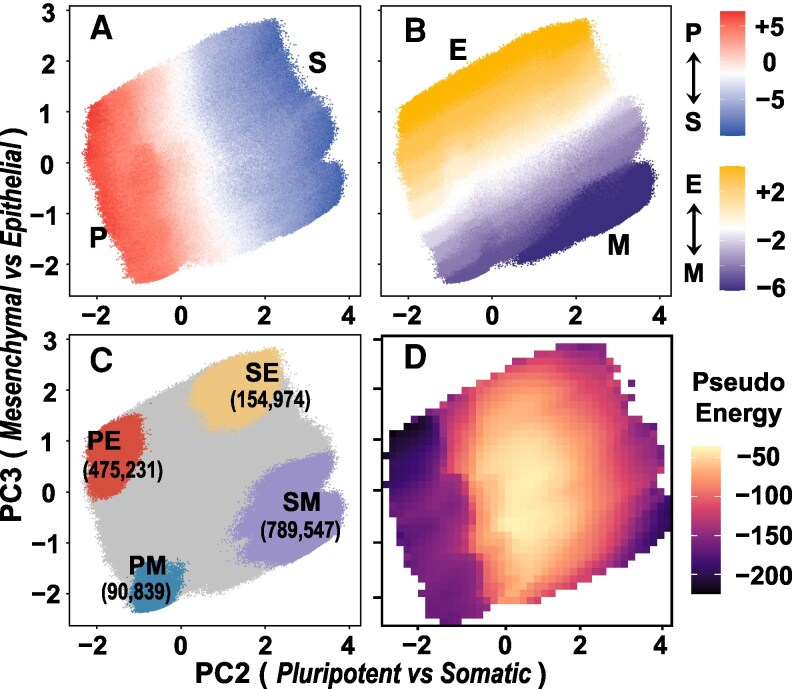
Phenotypic landscapes of reprogramming systems. A and B) Projection of the obtained $10^{7}$ steady states along the two essential collective variables PC2 and PC3 from principle component analysis. The steady states are colored according to the phenotypic scores $\Phi_{\mathrm{PS}}$ (A) and $\Phi_{\mathrm{EM}}$ (B). C) Same as A and B), but with the steady states colored according to their phenotypic scores, highlighting the top and bottom 20%. D) Averaged pseudo-energy of the steady states along the two essential collective variables PC2 and PC3.

We randomly generate $10^{7}$ initial states and simulate the relaxation dynamics to the steady states following Eq. 1. On average, the system took 32.7 steps to reach the final steady states. These steady states collectively form phenotypic phase space that quantify the two phenotypic properties as shown in the principle component analysis (Fig. 2A and B). Cell states located at the peripheral regions along the 2D essential space exhibit the most prominent phenotypic features (Fig. 2C). SM-/PE-like regions respectively act as the source (MEFs) and target (iPSCs) populations of reprogramming processes. These results suggest that successful cell fate transition relies on appropriately breaking and rebuilding cellular order. Therefore, it is crucially important to investigate the underlying dynamic and interaction characteristics of the gene regulatory network in cell fate transitions.

To quantify the consistency of the regulatory relationship between different genes, we calculated the cellular pseudo-energy following previous works (28–30), which is given by


(3) $$\tilde{E}(\vec{x})=-\sum_{\left\langle i,j\right\rangle }A_{ij}{\tilde{x}}_{i}{\tilde{x}}_{j}.$$

Here, ${\tilde{x}}_{i}$ takes the value of +1 and −1, respectively, for the active and inactive genes. The genetic pseudo-energy landscape along the 2D essential space shows that the cell states with significant phenotypes tend to have lower pseudo-energy (Fig. 2D). This result suggests that these states have well-orchestrated regulatory patterns and therefore low frustration, consistent with previous findings that gene regulatory networks for cell fate decision are minimally frustrated (29). In addition, the cell states locating between the significantly phenotypic states exhibit much higher pseudo-energies, which suggests that cell fate transition may involve a barrier-crossing feature. It is worth noting that the pseudo-energy defined above was introduced to quantify the consistency of the regulatory interactions among the nodes of the nongradient genetic networks. Therefore, the barrier on this pseudo-energy landscape is different from the barrier typically encountered in the thermally activated transitions along a physical energy landscape. In analogy with the typical physical systems, the barrier is featured by less populations of the cell states as shown in the steady-state simulations (Fig. S1). The high pseudo-energy around the barrier rises from inherent inconsistent regulatory relation of some states with inapparent phenotypes, which therefore are featured by high frustrations.

### Role of induced genetic frustrations in triggering cell fate transitions

**Fig. 3.**
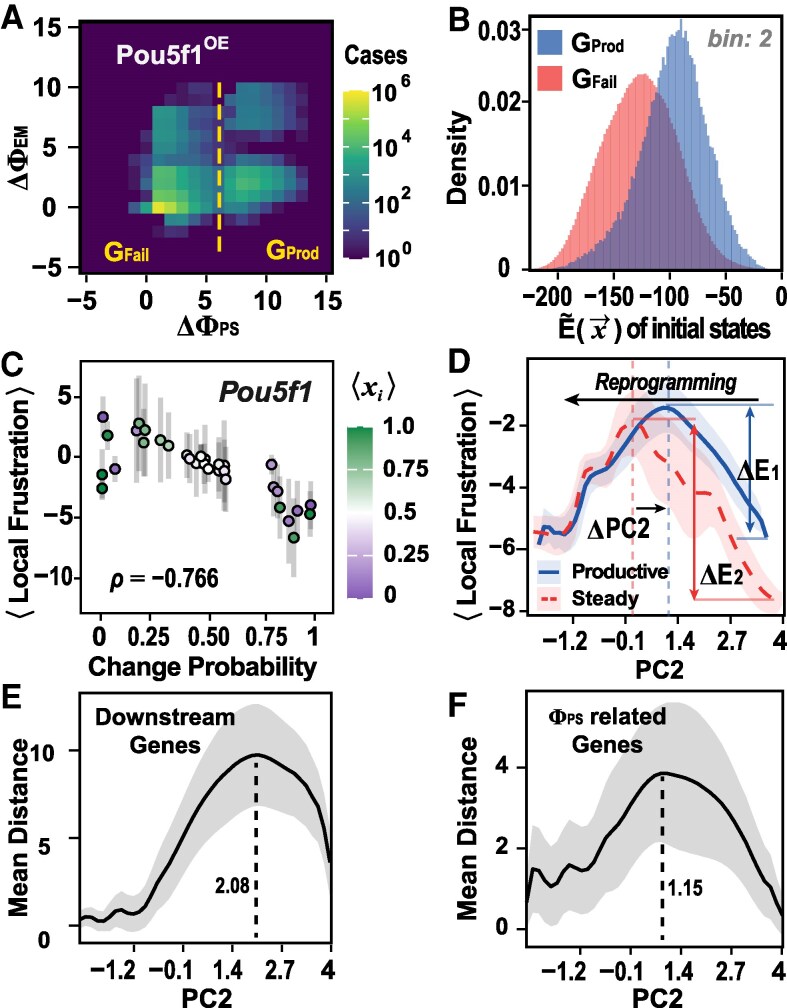
Role of local frustration in perturbation-induced cell fate transition from SM to PE states. A) Response of MEF-like cells to Pou5f1 overexpression. Simulation trajectories are categorized into two groups: with productive reprogramming ($\Delta \Phi_{\mathrm{PS}}>6$) or unproductive reprogramming ($\Delta \Phi_{\mathrm{PS}}<6$). The two groups are symbolized as $G_{\mathrm{Prod}}$ and $G_{\mathrm{Fail}}$, respectively. B) Distributions of pseudo-energies $\tilde{E}(\vec{x})$ for the initial states of the two groups of simulation trajectories. C) Correlation between the local frustration of different genes under Pou5f1 overexpression and their probabilities of have different gene states between the initial and final cell states. High-susceptibility genes tend to have lower local frustration. The color of the points represents the average expressions of genes in their initial states. *ρ*, Pearson correlation. D) Average local frustration for the high-susceptibility genes along PC2 after introducing Pou5f1 perturbation for the productive reprogramming trajectories (productive). For comparison, the averaged local frustration for the sampled steady states shown in Fig. 2 are also shown (steady). Introducing perturbation elevated the energies of the initial states, decreases the barrier height of cell fate transition, and shifts the location of the transition barrier. $\Delta E_{1}=3.11$, $\Delta E_{2}=4.25$, ΔPC2=0.65. Mean distance between the cell states for direct downstream targets of the perturbed gene (E) and genes associated with the $\Phi_{\mathrm{PS}}$ phenotype (F) during reprogramming, respectively.

Due to the low frustration of the intrinsic regulatory network, successful cell reprogramming always relies on external perturbations, such as adding specific transcription factors. These external perturbations often lead to the overexpression of specific genes, altering cell fates. Starting from the initial SM states, we performed extensive simulations following the scheme given in Eq. 1. Some trajectories can successfully reach the final PE state, corresponding to productive cell reprogramming events, while others fail to find the final PE state (Figs. 3A and S2). By comparing these two sets of trajectories, we may be able to infer the key features contributing to productive cell fate transition. Firstly, we calculated the pseudo-energy distributions for the initial states (sampled from the SM state) of the two sets of trajectories. Interestingly, the initial states for these productive trajectories are featured by much higher pseudo-energies, and therefore higher global frustration, compared to those of the unproductive trajectories. For example, the average pseudo-energies are −95.91 and −125.47, respectively, for the two sets of initial states (Figs. 3B and S3). These results suggest that sufficient frustration is essential for the initiation of the productive cell fate transition events.

Cell reprogramming is always accompanied with the alteration of activity states of some specific genes. By analyzing the difference of the genetic states between the PE and SM cell states, one can identify the susceptible genes subjective to alteration of activity during cell reprogramming (Fig. S4). Genes with a higher probability of changing their states are classified as high-susceptibility genes. In addition to analyzing the feature of global frustration of the gene regulatory network, it is also valuable to investigate the local frustration for each gene in the network. Similar to the pseudo-energies defined above for characterizing global frustration, we can quantify the local frustration by defining the pseudo-energies for individual genes, which is given by


(4) $${\tilde{E}}_{i}=-\sum_{\left\langle j\right\rangle }A_{ij}{\tilde{x}}_{i}{\tilde{x}}_{j}.$$

Where the summation runs over all the $N_{i}$ genes regulating gene *i*. Intriguingly, high-susceptibility genes tend to have much lower local pseudo-energy compared to other genes in initial cell state, regardless of whether they are expressed (Figs. 3C and S5). Therefore, the low frustration of these susceptible genes can be essential for maintaining the stability of cell states in a noising environment.

We then calculated the single-site pseudo-energy of the high-susceptibility genes for the snapshots sampled in the productive trajectories under Pou5f1 perturbation protocol along the essential reaction coordinate PC2 (Figs. S6 and S7). For comparison, we also calculated the single-site pseudo-energies for all the sampled steady states shown in Fig. 2. Similar to the global pseudo-energy landscape shown in Fig. 2D, the single-site pseudo-energy also exhibits a barrier-crossing feature (Figs. 3D and S8). It is important to emphasize again that the transition state discussed here for the cell fate decision is only conceptually in analogy with the transition state of thermally activated physical processes and therefore not necessarily corresponds to the rate-limiting step in the cell reprogramming. Moreover, Figs. 3D and S8 demonstrate that pseudo-energies of the snapshots along the reaction coordinate for the productive trajectories have higher local frustration at the initial state, leading to a reduced barrier for the cell reprogramming. Additionally, the location of transition state is shifted towards the initial SM state, suggesting a global effect of the external perturbation on the genetic regulatory landscape. These observations also corroborate the findings in Ref. (28), which showed that overexpression of gene SNAI1 leads to a tilt of the phenotypic landscape, favoring the epithelial–mesenchymal transition. These results suggest the crucial role of the induced local genetic frustrations in triggering cell fate transitions. Introducing external perturbations can induce local frustration in the high-susceptibility genes, which in turn reduces the barrier of the reprogramming transition and modifies the transition state. Although the overall genetic regulatory network for cell fate decision is minimally frustrated as revealed in previous works (29), local frustration induced by external perturbation is essential for successful cell reprogramming.

As discussed above, the states located around the barrier in the pseudo-energy profile have a high degree of disorder during reprogramming process and are less consistent compared to the initial and final phenotypic states. To further explore structural features of the barrier, we analyzed the heterogeneity of the expression profiles across various cell states along the same reaction coordinate. We calculated the distance between two cell states based on the activity states of specified genes by


(5) $$D(\vec{x},\vec{y})=\sum_{i}|x_{i}-y_{i}|.$$

Where $\vec{x}$ and $\vec{y}$ denote two cell states; *i* denotes a certain gene of observation. We then further calculated the mean values of the cell distances among the cell states at different range of reaction coordinate PC2. Higher values of mean distance suggest larger heterogeneity of the cell states. The results show that cell states around the barrier are much more heterogeneous compared to the initial and final states of the cell reprogramming (Fig. 3E and F). Such a features of high heterogeneity is consistent with the transition state during cell fate decisions discussed in Refs. (36–39). The reorganization of genetic information can result in critical behaviors and amplify the heterogeneity of genetic profiles.

The above results are reminiscent of the role of energetic frustration in proteins as elucidated by the energy landscape theory of protein folding (40). This framework posits that evolution has optimized foldable protein sequences to minimize energetic frustration in their native states, thereby ensuring efficient folding kinetics and structural fidelity. Meanwhile, localized frustration emerges as an evolutionary adaptation at functionally critical regions (41). Particularly, in many allosteric proteins, for which the functioning dynamics often involve the switching between different conformational states, the residues subjective to conformational motions are often locally frustrated (42–45). The identified role of localized frustration within genetic regulatory networks demonstrates remarkable functional universality with these evolutionarily refined mechanisms in natural biosystems.

The frustration discussed above reflects the inconsistency between gene states and regulatory patterns. With the progression of cell reprogramming, the cellular order needs to re-establish, and the driving force arising from such inconsistency is expected to decrease gradually. To quantitatively quantify such effect, we defined the driving force as following


(6) $$\mathrm{DF}(t)=\sum_{i}|x_{i}(t)-\Theta (\sum_{j}A_{ij}x_{j}(t))|.$$

**Fig. 4.**
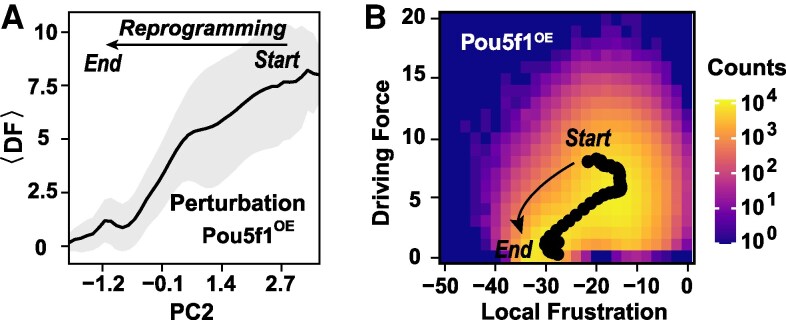
Driving force features during reprogramming. A) The average transient driving force ⟨DF⟩ decreases throughout the reprogramming process. B) The heatmap illustrates the relationship between high-susceptibility genes local frustration and driving force. Each bin records the count of transient cells that pass through this bin across all productive paths. Black points denote mean values along the reaction coordinate (PC2). The color bar indicates numerical ranges.

Here, the index *j* runs over all the genes regulating the gene *i*; *Θ* denotes the dynamic rule given in Eq. 1. As expected, the driving force has a clear decreasing trend as the cell state evolves from the starting somatic state to the final pluripotent state during the reprogramming (Fig. 4A), showing re-establishment of new cellular order. With the progression of reprogramming, the variance of DF also tends to decrease, possibly because of the decreasing of cell heterogeneity during the later stage of reprogramming (Fig. 3E and F).

According to the definition given by Eq. 6, DF describes the variation potential of the gene states, therefore it is related to the local frustration, which characterizes the inconsistency between gene states and regulatory patterns. To elucidate the relationship, we plotted the reprogramming pathway along the DF and local frustration (Fig. 4B), which roughly shows a positive correlation due to the constraints of the rule *Θ*. The reprogramming processes proceed along complex paths rather than directly from initial states to final states (Fig. S9), reflecting the complexity of the underlying regulatory structure.

### Scale-free pattern of the induced cell fate transitions

Yamanaka and other induction protocols utilize a limited number of factors to induce cell reprogramming. Detailed analysis shows that these factors tend to induce more significant local frustration (Fig. S7), as evidenced by substantial changes in single-site pseudo-energy. To assess the order-reshaping potential of each gene, we performed simulations by introducing overexpression (OE) and knockdown (KD) to the genes of the regulatory network. This was realized by setting the genetic states of the corresponding genes to 1 and 0, respectively, throughout the whole simulation trajectories. The simulation starts from a random initial cell state. The results show that OE of different gene has different effect on the variation of the genetic states of the final steady states. To more quantitatively characterize such effect, we calculated the Hamming distance of expression profiles between the initial cell state and the final steady state from the simulations, which is given by,


(7) $$\Delta (\vec{x})=\left\|{\vec{x}}_{initial}-{\vec{x}}_{\;final}\right\|,$$

where ${\vec{x}}_{initial}$ and ${\vec{x}}_{\;final}$ are the genetic states at the initial and final snapshots, respectively, of the simulation trajectory. It corresponds to the total number of genes which have different states at the initial and final cell states. A larger *Δ* value suggests that the perturbation of a certain gene has a more pronounced effect on the cell fate transition.

**Fig. 5.**
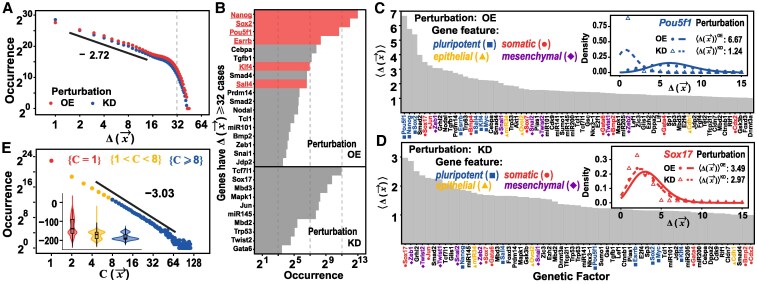
Scale-free pattern of the induced cell fate transitions. A) The distributions of the Hamming distance $\Delta (\vec{x})$ for all the OE/KD simulation trajectories. The simulations are initiated from each of the steady states shown in Fig. 2 but with introducing OE (red) or KD (blue) for one of the genes. The repetitive steady states are excluded. B) Occurrence of the events with $\Delta (\vec{x})⩾32$ for overexpressing different genes. Only the genes with the occurrence larger than 10 are plotted. Genes marked in red and underlined are classic genetic factors commonly used via overexpression in reprogramming experiments. C and D) The effects of individual gene OE/KD perturbations on cellular state alteration. Genes are ranked by $\left\langle \Delta (\vec{x})\right\rangle$, which is quantified using fitted Poisson distributions under OE/KD perturbation. Some classical genes associated with the four phenotypic features are marked by corresponding colors and symbols as illustrated in legends. Only genes $\langle \Delta (\vec{x})\rangle >1$ are presented here. Inset figures show two examples of classical pluripotent (Pou5f1) and somatic (Sox17) genes. E) Distribution of the cell-state-capacity $C(\vec{x})$ among the steady states shown in Fig. 2. The pseudo-energy distributions of the steady states with different $C(\vec{x})$ with are also shown as inset.

Figure 5A shows the distribution of *Δ* from different simulation trajectories. Interestingly, the *Δ* distribution exhibits a power law pattern over a wide range of *Δ* values and phenotypic features. Such scale-free patterns suggest that there exist a small number of genes for which OE has the most significant effect on cell phenotypic change. The genes contributing to the heavy tail of the power law distribution may provide potential clues for important regulatory patterns. When examining cases with Δ⩾32, it is observed that these genes predominantly belong to TFs such as Nanog, Sox2, Pou5f1, Esrrb, Klf4, and Sall4, which are associated with maintaining pluripotency (Fig. 5B) (2–12). In addition, the distribution of $\Delta (\vec{x})$ values for these genes demonstrate the significant effect of these genes on altering cell states. The results also showed that OE interventions are more effective than KD ones, which is in line with previous experimental observations that overexpressing some TFs instead of suppressing them is more effective in inducing cell reprogramming (Fig. 5C and D).

We next analyzed the features of the final cell states. For each steady state $\vec{x}$, we defined the cell-state-capacity $C(\vec{x})$, which is given by the number of initial random cell states leading to the final steady state $\vec{x}$ in the simulations. The $C(\vec{x})$ distribution also shows a scaling-free pattern, indicating that majority of the final steady states accommodate only a limited number of initial states. In contrast, a small number of steady state can accommodate a large number of initial states. These large domains of attraction often have lower pseudo-energy, indicating that ordered cell states demonstrate stronger dynamic attraction (Fig. 5E).

### Experimental data validation

**Fig. 6.**
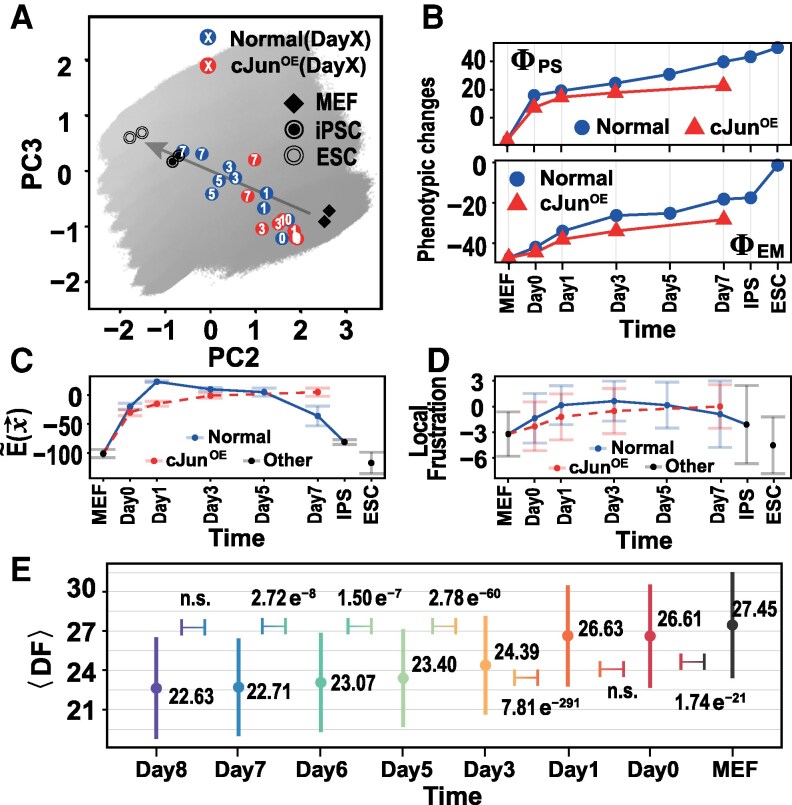
Experimental data showing the changes in various genetic features during somatic cell reprogramming under Yamanaka protocols. A) Cell reprogramming trajectories from experiments projected along the essential space formed by PC2 and PC3 for the normal Yamanaka protocol and for the Yamanaka protocol with cJun^OE^. MEF, iPSC, and ESC represent mouse embryonic fibroblasts, induced pluripotent stem cells, and embryonic stem cells, respectively. The background landscape is similar as that shown in Fig. 2, which only common genes are considered. Note that absolute distances and locations are intended solely as reference, serving to reflect general trends. B) Phenotypic scores calculated from experimental data along the time series of cell reprogramming with different protocols. Pseudo-energies C) and local frustrations D) along the time series of cell reprogramming for the two experimental protocols. Tag *Other* indicates cells that are not on the reprogramming trajectories, such as MEFs, iPSC, and ESC for reference. E) Averaged driving force ($\mathrm{DF}(\vec{x}(t))$) during cell reprogramming from single-cell mRNA-Seq data. The symbols and vertical lines represent the means and standard deviations at each time point. The *p*-values from t-test showing the significance of the difference between two adjacent distributions are also labeled. To keep consistency with Fig. 3A, the cell reprogramming progresses from right to left.

As a further demonstration of the crucial role of local frustration in cell fate transition, we analyzed the dynamic and energetic features of mRNA sequencing data (mRNA-Seq) collected in cell reprogramming experiments. In a previous experimental work, Li et al. employed the standard Yamanaka protocol to implement reprogramming (3). As a control, an additional experiment was conducted by introducing overexpression of cJun (cJun^OE^). cJun is a transcription factor that protects the epigenetic state of somatic genes but weakens the epigenetic activity of pluripotent genes. To quantify the time series of the cell fate transition, we binarized the bulk mRNA-Seq and embedded it into the phenotypic diagram. For the canonical Yamanaka protocol, successful cell state transition from MEF-/SM-like state to iPSC-/PE-like state were observed (Fig. 6A). In contrast, the cJun^OE^ cases are limited around the initial points, failing to progress from MEF-/SM-like to iPSC-/PE-like regions. Moreover, continuous phenotypic scores also indicate that intermediate trajectories are blocked by abnormal genetic status caused by cJun^OE^ (Fig. 6B).

We then calculated the instantaneous energies of the cell states collected after different time lags in the above two experiments based on the measured genetic activity states. Consistent with the above simulation data, we observed the nonmonotonic change of the pseudo-energy with the progression of cell reprogramming for the Yamanaka protocol experiment. The pseudo-energy increases initially and then decreases over time, which indicates that cell reprogramming involves a energy barrier (Fig. 6C). In contrast, for the cJun^OE^ case, the pseudo-energy increases initially and then becomes saturated, lacking an order-reshaping phase essential for cell reprogramming. We also analyzed the local frustrations of various susceptible genes. The local frustration also shows nonmonotonic change similar to the time dependence of the global pseudo-energy for the Yamanaka protocol (Fig. 6D).

To further quantify the role of genetic frustration during the cell reprogramming procedure, we analyzed the distributions of the DF values from the single-cell mRNA-Seq with the Yamanaka approach (7), observing a similar evolutionary pattern as seen in the simulation data of the productive reprogramming events (Fig. 3E). The mean value of the DF consistently decreases with the progression of the reprogramming (Fig. 4A), which signifies the re-establishment of the new cellular order towards target cells.

## Discussion and conclusion

Cell fate transitions are complex processes orchestrated by established blueprints—regulatory relations. In this study, we consider somatic cell reprogramming processes as paradigmatic cases to capture the key role of genetic frustrations on phenotypic reshaping by performing numerical simulations and cell reprogramming experiments. The results showed that genetic frustrations plays a key role in inducing the cell reprogramming, although the regulatory network are overall minimally frustrated. Critical minority genes within systems contribute to phenotype-oriented perturbations, inducing significant genetic frustrations. In biological scenarios, the extreme inefficiency of transitions indicates inherent obstacles within cells beyond the “blueprints.” The inducing factors, perturbation duration, and epigenetic status collectively complicate the development of universal schemes for controlling cell fate transitions. The fundamental principles of inducing protocols necessitate preparing epigenetic modifications, regularizing transcriptome of starting cells, and creating genetic status of targeted cells (46, 47). All of these strategies are practically designed to effectively trigger and influence local genetic frustrations. The key role of genetic frustration also underscores the significant genetic inertia of cells, reflecting the role of cellular memory in cell fate decisions (48, 49). Note that the model frameworks and numerical approaches employed here are preliminary and oversimplified, lacking the complex coupling relations. In future investigations, more precise models, potentially incorporating other omics (50, 51), are required to provide a clearer understanding of genetic landscapes, thereby more accurately describing cellular states and measuring changes in genetic frustrations. Such an approach will contribute to elucidating the universal principles governing cell fate transitions.

In this work, we demonstrated that the cell states around the barrier position in the pseudo-energy landscape are much more heterogeneous than the initial and final states of the cell reprogramming. Such result may suggest that cell reprogramming involves an intermediate stage with enhanced heterogeneity, therefore it needs to explore a wider range of cell states along the phase space. Interestingly, previous studies have demonstrated that a key prerequisite for regulating genome expression is its unique position at the edge of chaos (36–39). Near critical points in the system, gene expression variability tends to increase, while correlations among certain genes diminish. Particularly, the overall uncertainty of the system rises, expanding the system’s global search space. The results of these works demonstrated an entropic reservoir mechanism (52). The high heterogeneity of the transition state identified in the simulations of this work is consistent with the entropic reservoir theme discussed before. In addition, the scale-free behavior of the induced cell fate transitions revealed in this work is in line with the self-organization mechanism discussed in previous works.

## Methods

### Simulation

The system evolution employed an asynchronous rule, wherein only one component is randomly selected to update its state at each time step. No gene has priority over others in the process of selection. To enhance computational efficiency, only those genes that are not yet steady can be selected for updating. Namely, the candidate gene *i* shall satisfy,


(8) $$\left\{ \begin{array}{c} \sum A_{ij}x_{j}>0\wedge x_{i}=0\\ \sum A_{ij}x_{j}<0\wedge x_{i}=1. \end{array}\right.$$

$\vec{x}(t+1)=\vec{x}(t)$ indicates that the system has reached a steady state. No limit cycles were observed in the simulations.

### Phenotypic markers

To represent the tendency during reprogramming, we specified final and start features as “positive” and “negative” classes, respectively. Table 1 displays the four clusters of phenotypic feature utilized in our analysis. The cells were treated as phenotypically significant if both of their scores rank in the top/bottom 20% (colored cell in Fig. 2C).

**Table 1.** Marker genes of phenotypes.

$\phi_{\mathrm{PS}}^{-}$ (S)	Gata6, Gata4, Bmp2, Bmp4, Cebpa, Jun, Sox17,
	Sox7, Cdx2, Foxa1, Foxa2
$\phi_{\mathrm{PS}}^{+}$ (P)	Esrrb, Nanog, Klf4, Pou5f1, Sox2, Myc, Sall4
$\phi_{\mathrm{EM}}^{-}$ (M)	Vim, Cdh2, Zeb1, Zeb2, Foxc2, Snai1, Snai2,
	Twist1, Twist2, miR9
$\phi_{\mathrm{EM}}^{+}$ (E)	Cdh1, Tcf3, Ovol2, miR200, miR34

### Principal component analysis and reaction coordinate

To show the phenotypic landscape, the stable states of system were analyzed via principal component analysis (PCA), whose cells were measured by two phenotypic scores. Then, $\Phi_{\mathrm{PS}}$ and $\Phi_{\mathrm{EM}}$ were smoothed using a *k*-nearest neighbor (KNN) averaging approach implemented through R library RcppHNSW with parameter k=5 (53). This methodology was based on the empirical observation that phenotypic features tend to be similar when they are in close proximity within phase space (28). In other cases, analyses were based on the actual values. Given that the acquisition of pluripotency is a significant event of reprogramming, we used the $\Phi_{\mathrm{PS}}$ (PC2), as the phenotypic reaction coordinate to observe cellular genetic changes. All intermediate states were projected onto the PC2 using the above PCA system, and the mean values were analyzed within specific bin sizes.

### Gene perturbation and changed genetic features

Overexpression (OE) and knockdown (KD), commonly used in biological experiments, serve as genetic perturbations, where perturbed genes are assigned constant values (1 for OE; 0 for KD). For simplification, we employ the symbol $\vec{\epsilon }$ as specific perturbed occasions, such as $\vec{\epsilon }\;:\;\{\epsilon_{i}=1\}$ for OE of gene *i*, $\vec{\epsilon }\;:\;\{\epsilon_{\mathrm{Pou}5f1}=1$, $\epsilon_{\mathrm{Sox}2}=1$, $\epsilon_{\mathrm{Klf}4}=1$, $\epsilon_{\mathrm{Myc}}=1\}$ for standard Yamanaka protocol. The controlled gene (CG) is fixed at a specific value, so its dynamic behavior is ignored. Consequently, at each simulation step, only unstable genes except for {CG} are considered. The external perturbation is not removed at the end of the simulations to show their genetic effects.

When attaining new steady states, perturbed cells also change their phenotypes and genetic characteristics. The alterations of phenotypes is calculated by,


(9) $$\left\{ \begin{array}{c} \Delta \Phi_{\mathrm{PS}}=\Phi_{\mathrm{PS}}({\vec{x}}_{\;f})-\Phi_{\mathrm{PS}}({\vec{x}}_{s})\\ \Delta \Phi_{\mathrm{EM}}=\Phi_{\mathrm{EM}}({\vec{x}}_{\;f})-\Phi_{\mathrm{EM}}({\vec{x}}_{s}). \end{array}\right.$$

The Hamming distance of expression profiles between start cells (${\vec{x}}_{s}$) and final cells (${\vec{x}}_{\;f}$) serves as an indicator of altered global genetic characteristics. It is defined by,


(10) $$\Delta (\vec{x})=\|{\vec{x}}_{s}-{\vec{x}}_{\;f}\|.$$

We use these two features to assess the effects induced by perturbations.

### Sequencing data

The sequencing data from the two experiments were categorized into bulk mRNA sequencing (GSE93027) (3) and single-cell mRNA sequencing (GSE103221) (7). The detail information can be found in original papers and the Gene Expression Omnibus (GEO) websites. We first obtained the intersection of the genes in the data with those present in our model. Then, we performed binarization using Gaussian Mixture Model and calculated the phenotypic scores based on the respective expression states. We generated a new PCA projection using the intersecting genes, which thus Fig. 6A differs slightly from Fig. 2. Due to the drop-out issue in single-cell sequencing technology, we initially screened for effective cells, considering only those cells whose number of expressed genes falls within the 5th to 95th percentile. Subsequently, we calculated their respective driving forces by definition.

## Supplementary Material

pgaf303_Supplementary_Data

## Data Availability

The data used in this article were available in Gene Expression Omnibus, at [https://www.ncbi.nlm.nih.gov/geo/], and can be accessed with [GSE93027, GSE103221]. This study did not generate new sequencing data. Other information are included in Supplementary material. Codes are available at https://github.com/YuxiangYao/GeneFrustration.

## Preprints

This manuscript was posted as a preprint, BiorXiv:2025.01.31.635856.
