# Supplementary material for: Role of frustrations in cell reprogramming
Source: PNAS Nexus. 2025 Sep 22;4(10):pgaf303. doi: 10.1093/pnasnexus/pgaf303 (PMC12487780; doi:10.1093/pnasnexus/pgaf303)
Supplement: pgaf303_Supplementary_Data [file pgaf303_supplementary_data.zip › PNASNEXUS-PNASNEXUS-2025-00656R-s01.pdf]

Supplementary figures for
“Role of Frustrations in Cell Reprogramming”

Yuxiang Yao, Jieying Zhu, Wenfei Li, Duanqing Pei

Figure S1: Distribution of the steady states in Figure 2 along the two essential variables PC2 and PC3.

Figure S2: Response of MEF-like cells to overexpressing reprogramming-related genes.

Figure S3: Initial frustration distributions of productive and failed groups.

Figure S4: Differences of all genetic factors (genes and miRNAs) between PE and SM region.

Figure S5: The correlations between alterations in gene expression rate and local frustrations.

Figure S6: Frustrations of the two types of trajectories vary with reaction coordinates.

Figure S7: Different transient local frustrations (LFs) of HSGs and LSGs.

Figure S8: LF barrier’s drift and reduction of productive trajectories.

Figure S9: Heatmaps of relationship between high-susceptibility genes local frustration and driving force.

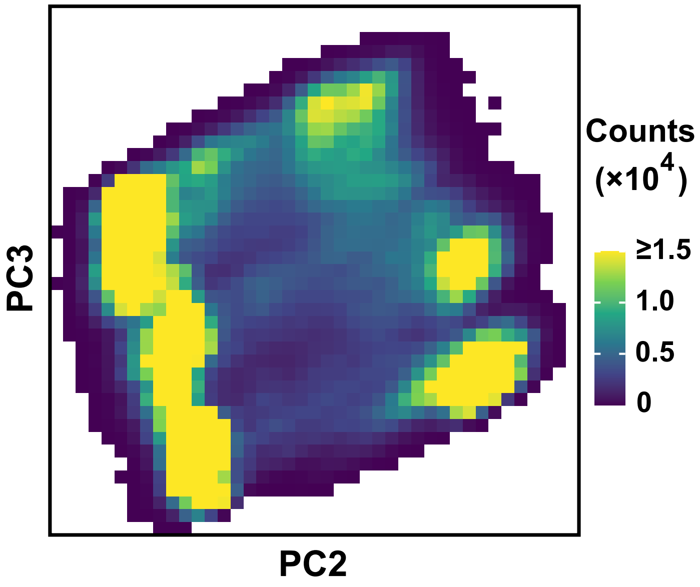

Figure S1: Distribution of the steady states in Figure 2 along the two essential variables PC2 and PC3. The color bar denotes numerical ranges. To mitigate the impact of a small number of extreme values, we set the upper bound at 15,000.

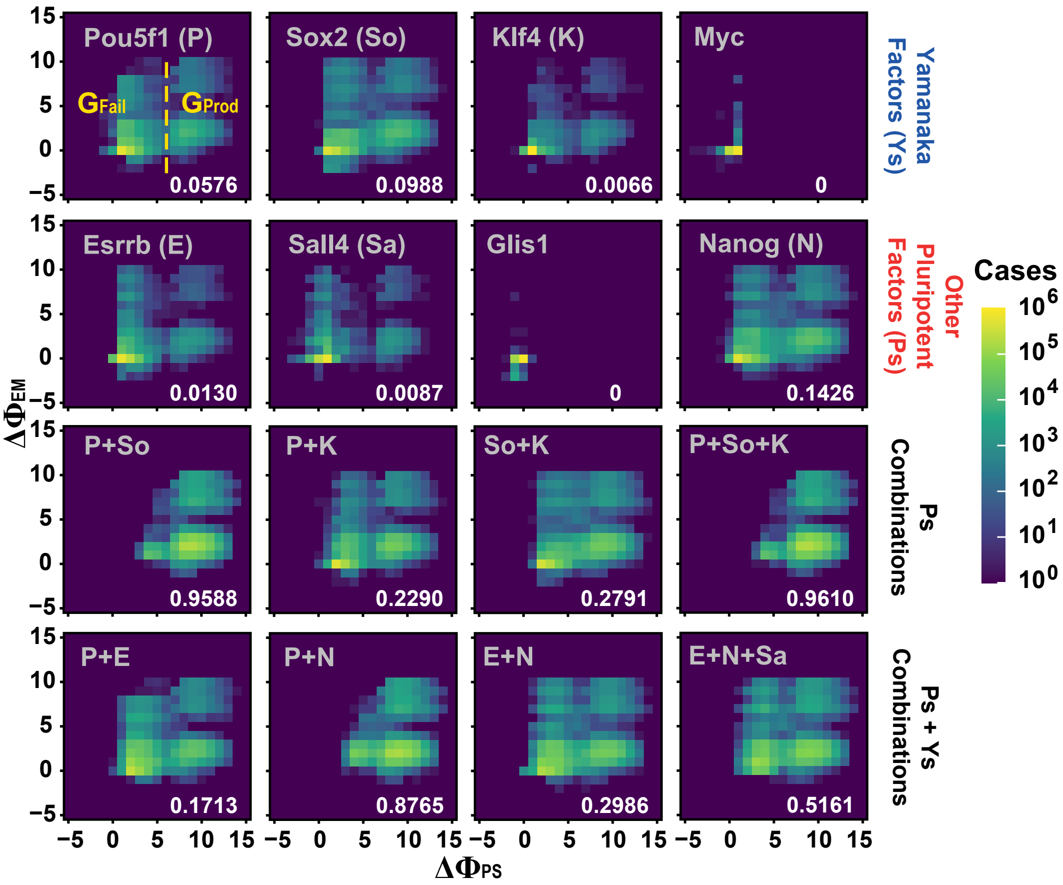

Figure S2: Response of MEF-like cells to overexpressing reprogramming-related genes. 10^6 random SM-feature steady states were regarded as initial cells and received overexpressing perturbations. As exemplified in Pou5f1 case, to simplify our analysis, $\Delta\Phi_{\mathrm{PS}} = 6$ categorize states as productive and failed cell reprogramming events, denoted by $\mathrm{G}_{\mathrm{Prod}}$ and $\mathrm{G}_{\mathrm{Fail}}$, respectively. Subsequent analyses also rely on this criterion. Numbers labeled in the corner denote the proportion of $\mathrm{G}_{\mathrm{Prod}}$. Four non-Yamanaka factors are selected from Ref[4] in main text.

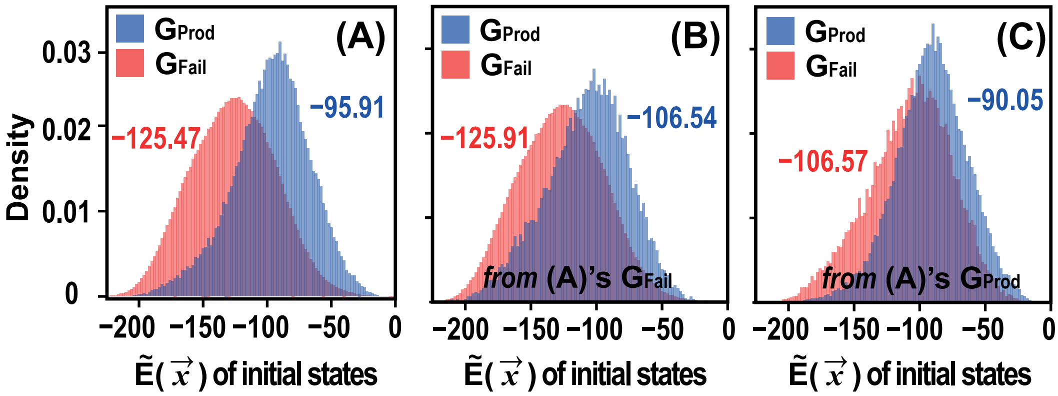

Figure S3: Initial inconsistency distributions of productive and failed groups. (A) Distributions of inconsistency CI($\vec{x}$) for the initial states of the two groups of simulation trajectories. (B,C) Reanalyzed distributions of cells of G_{Fail} and G_{Prod}, respectively. Colored numbers denote the average frustrations. Bin width of bars is 2.

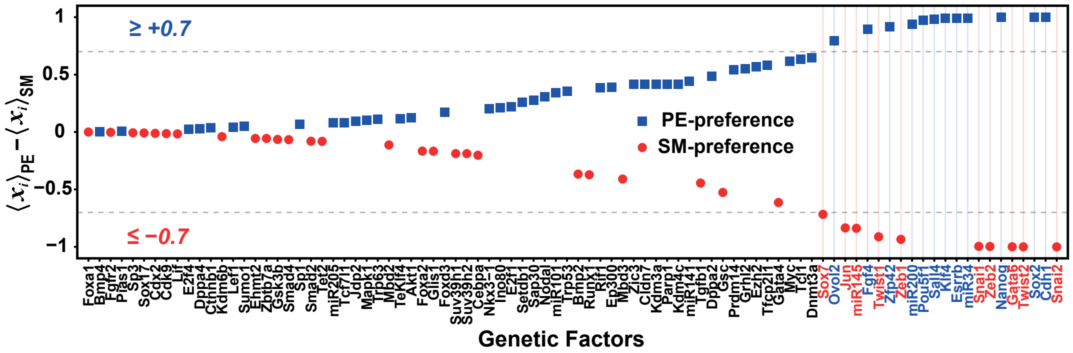

Figure S4: Differences of all genetic factors (genes and miRNAs) between PE and SM regions. The values range between $[-1, +1]$, with values closer to $+1$ (-1) indicating a more general expression within the PE (SM) type. By definition, positive and negative values denote PE- and SM-preference expression, respectively. Horizontal dashed lines distinguish high and low susceptibility genes based on ± 0.7. Genes with high susceptibility are labeled in color.

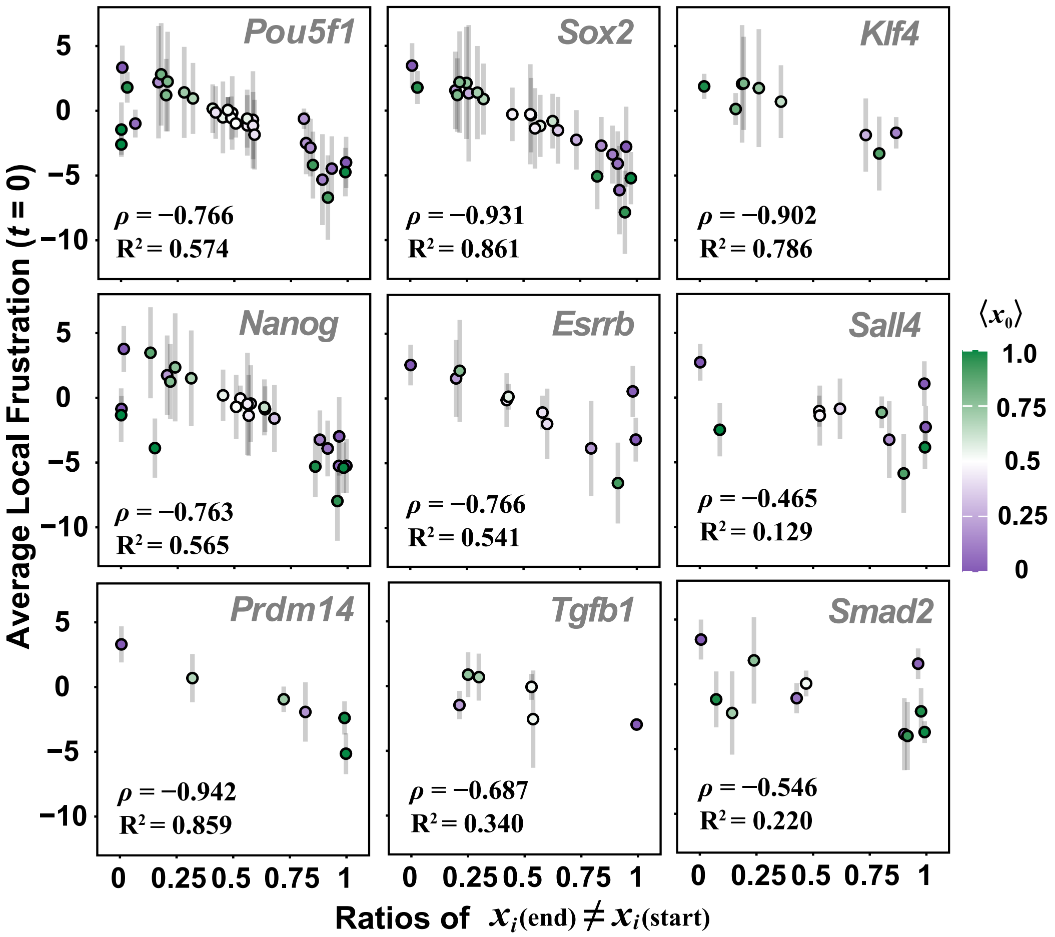

Figure S5: The correlations between alterations in gene expression rate and local frustrations. Only those perturbations with numerous genes are displayed here. Pearson correlation (ρ) and adjusted R-squared (R^2) of linear fitting are labeled in each subfigure.

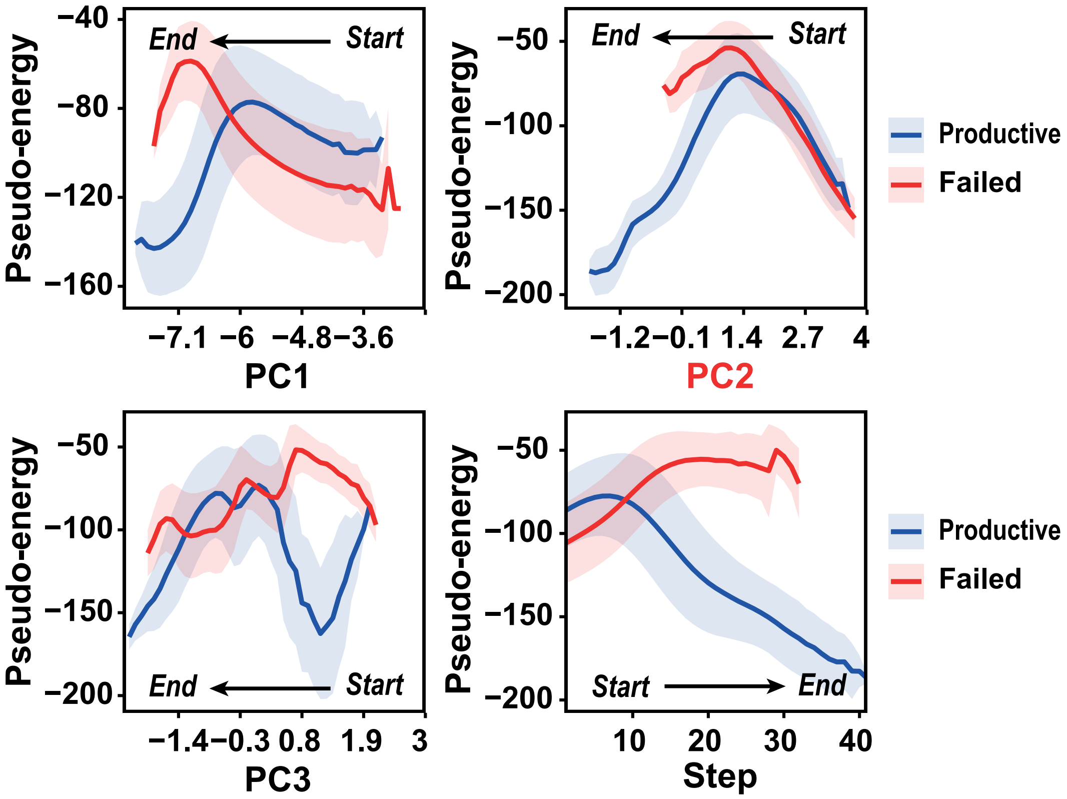

Figure S6: inconsistencies of the two types of trajectories vary with reaction coordinates PC1∼PC3 of figure 2 in main text, and simulated steps. The bin size of each PC is 0.02. Arrows from *Start* to *End* denote the directions of processes. The examples are the snapshots sampled under Pou5f1 OE perturbation.

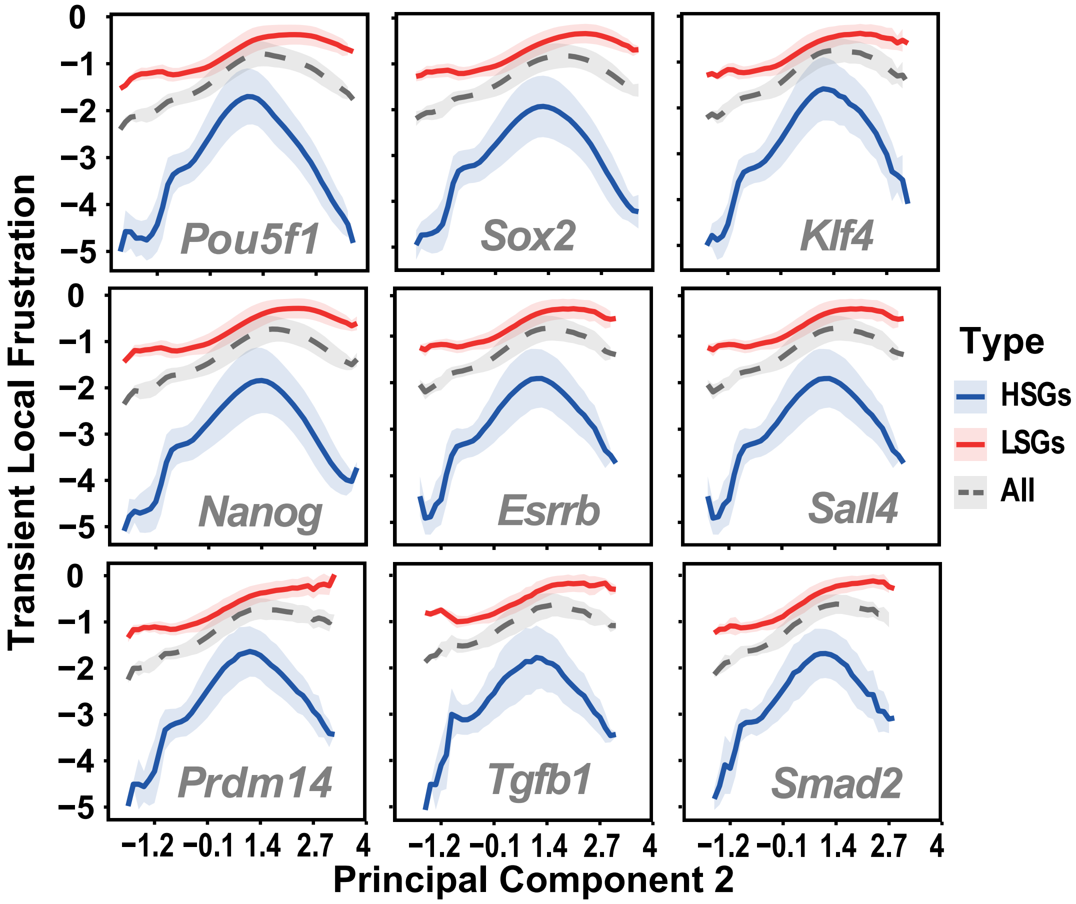

Figure S7: Different transient local frustrations of HSGs and LSGs. The bin size of PC coordinate is 0.02. All curves are productive processes.

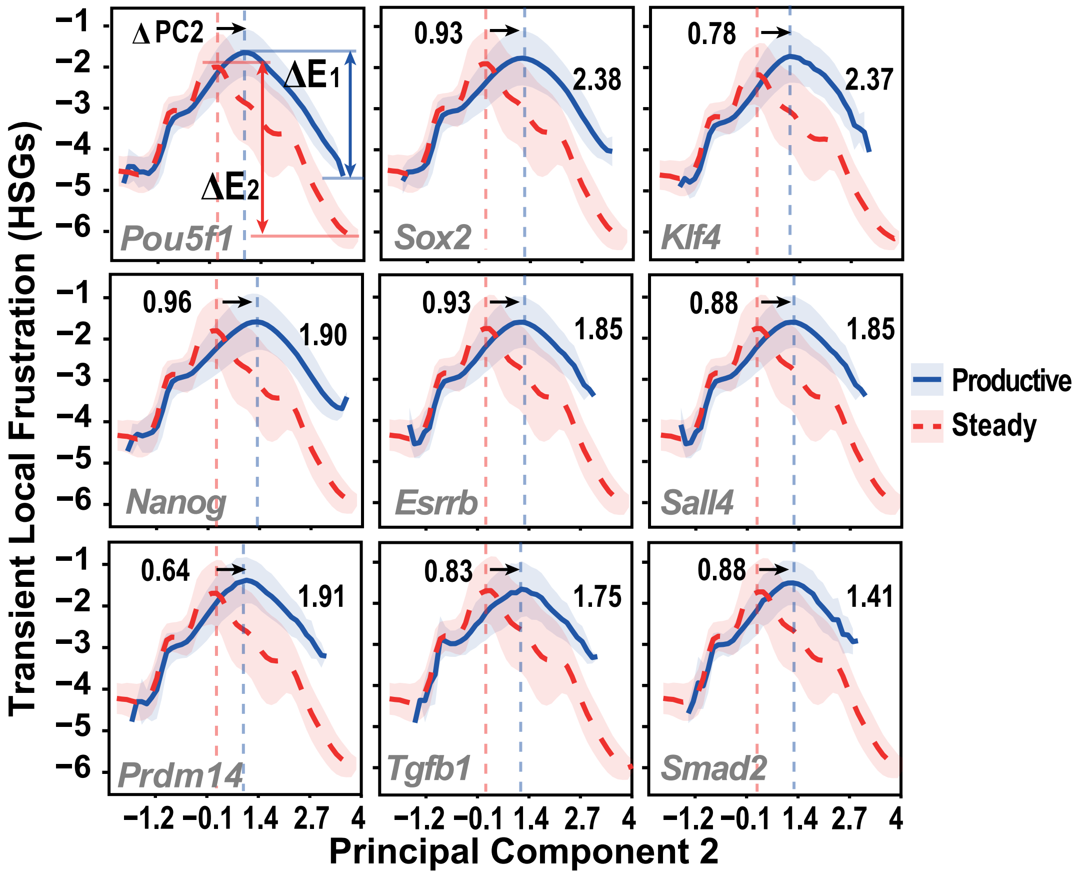

Figure S8: LF barrier's drift and reduction of productive trajectories. Dashed lines denote corresponding peaks. Relevant definitions are shown in Pou5f1-subfigure. ΔE_1, ΔE_2 denote the barriers of productive trajectories and steady states. Thus ΔE_2 remains a constant value of 4.25. ΔPC2 means the drift of peak location. ΔE_1 and ΔPC2 are marked in the upper left and upper right corner of each subfigure. Reprogramming direction is from right to left. The bin size of PC coordinate is 0.02.

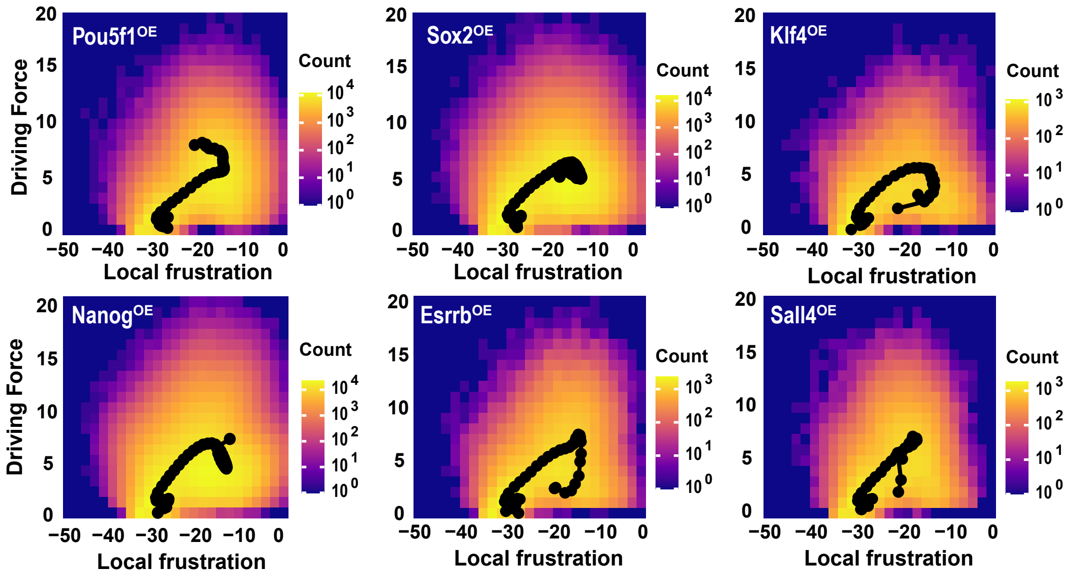

Figure S9: Heatmaps of relationship between high-susceptibility genes local frustration and driving force. Each bin records the count of transient cells that pass through this bin across all productive paths. Black points denote mean values along the reaction coordinate (PC2). The color bar indicates numerical ranges.
